# Incidence, etiologies, and outcomes of severe pediatric community-acquired empyema before and after the pandemic: an Italian multicentric study

**DOI:** 10.1007/s00431-025-06411-2

**Published:** 2025-09-04

**Authors:** Danilo Buonsenso, Carlotta Montagnani, Lorenza Romani, Anna Camporesi, Lucia Scarlato, Marco Denina, Daniele Zama, Aldo Naselli, Sonja Montonesi, Ilaria Liguoro, Marcello Mariani, Fabio Cardinale, Elena Chiappini, Maia De Luca, Silvia Garazzino, Samantha Bosis, Giulia Pattarino, Valentina Burzio, Luca Di Napoli, Claudia Colomba, Giangiacomo Nicolini, Giulia Lorenzetti, Luisa Galli, Andrea Lo Vecchio, SITIP Empyema Working Group

**Affiliations:** 1 Pediatric Infectious Diseases, Pediatric Emergency Care and Pediatric Ultrasound Department of Woman and Child Health and Public Health and Fondazione, Policlinico Universitario “A. Gemelli,” , Rome, Italy; 2 Pediatric Infectious Diseases Unit, Meyer Children’s Hospital IRCCS, Florence, Italy; 3 Infectious Diseases Unit, Bambino Gesù Children’s Hospital, IRCCS, Rome, Italy; 4 Infectious Diseases Unit, Department of Pediatrics, University of Turin, Regina Margherita Children’s Hospital, Turin, Italy; 5 Department of Medical and Surgical Sciences, Alma Mater Studiorum, University of Bologna, Bologna, Italy; 6 Pediatric Emergency Unit, IRCCS Azienda Ospedaliero-Universitaria Di Bologna, Bologna, Italy; 7 Neonatal Intensive Care Unit, Transmural Pediatric Department, S. Chiara Hospital, Trento, Italy; 8 Department of Pediatrics, Hospital of Bolzano, Bolzano, Italy; 9 Division of Pediatrics, University Hospital of Udine, Udine, Italy; 10 Pediatric Infectious Diseases Unit—IRCCS Istituto Giannina Gaslini, Genoa, Italy; 11 Department of Pediatrics, Pediatric Hospital Giovanni XXIII, University of Bari, Bari, Italy; 12 Department of Health Sciences, University of Florence, Florence, Italy; 13 Fondazione IRCCS Ca’ Granda Ospedale Maggiore Policlinico, Milan, Italy; 14 Department of Mother and Child Health, Division of Pediatrics, ASST Grande Ospedale Metropolitano Niguarda, Milan, Italy; 15 Department of Health Sciences, Division of Pediatrics, University of Piemonte Orientale, Novara, Italy; 16 Arcispedale Sant’Anna, University Hospital of Ferrara, Ferrara, Italy; 17 Department of Health Promotion, Mother and Child Care, Internal Medicine and Medical Specialties, University of Palermo, Palermo, Italy; 18 Pediatric and Neonatal Pathology Unit, Medical Area Department, S. Martino Hospital, Belluno, Italy; 19 Department of Translational Medical Sciences, University of Naples Federico II, Naples, Italy; 20 S. Spirito Hospital, Azienda Sanitaria Locale, PediatricsPescara, Italy; 21 Azienda Ospedaliero Universitaria Policlinico Di Modena, Modena, Italy; 22 Department of Pediatrics, University of Chieti-Pescara, Chieti, Italy; 23 Pediatria E Neonatologia, Ospedale Veris Delli Ponti Di Scorrano, ASL Lecce, Lecce, Italy; 24 NEUROFARBA Department, University of Florence, Florence, Italy; 25 Pediatric Unit, Meyer Children’s Hospital IRCCS, Florence, Italy; 26 Pediatric Emergency Department, Regina Margherita Children’ S Hospital, Turin, Italy; 27 Area Pediatrica, Dipartimento di Scienze della Vita e Sanità Pubblica, Università Cattolica del Sacro Cuore, Roma, Italia

**Keywords:** Empyema, Pneumonia, Children, COVID-19, Pandemic, SARS-CoV-2

## Abstract

An increase in severe and invasive infections has been reported since the COVID-19 pandemic. However, most evidence comes from monocentric studies without nationwide representativeness. This multicenter, nationwide, retrospective study, conducted within the network of the Italian Society of Pediatric Infectious Diseases (SITIP), aimed to compare the severity of empyema at presentation in children (aged 1 month to 18 years) admitted to 19 Italian hospitals before, during, and after the pandemic. A severe composite outcome was defined as either fatality, need for respiratory support or thoracic surgery, or admission to the pediatric intensive care unit (PICU). Among the 266 patients included in the study (38.7% females, median age of 4 years [IQR 2–7 years]), 95 (35.8%) were reported during the pre-COVID phase, 32 (12.1%) during the COVID phase, and 138 (52.1%) during the post-pandemic phase. The incidence of empyema significantly increased during the post-pandemic phase (pre-COVID: 95/19,288,639 [0.49]; during COVID pandemic: 32/18,784,272 [0.17]; post-pandemic: 138/18,294,627 [0.75]; *p* = 0.001). No differences in the demographic characteristics between the three groups were noticed, but a statistically significant difference was detected in the severe composite outcome (*p* = 0.029), as well as in the clinical (*p* = 0.006) and laboratory (*p* = 0.015) disease severity in children admitted during and after the pandemic. An increased odds of severe outcomes was observed during the COVID period (OR: 3.428, 95% CI: 1.21–9.65, *p* = 0.020) and in patients with complicated effusion observed at lung ultrasound (OR: 3.29, 95% CI: 1.26–8.57, *p* = 0.015). Each day of persistent fever was associated with a 10% increased risk of severe outcome (OR: 1.10, 95% CI: 1.03–1.18, *p* = 0.004). Since the onset of the pandemic, we observed an increased use of high flow nasal cannula.

*Conclusion*: Our analysis of children admitted in Italy confirms a surge in the incidence of empyema and an increase in disease severity during and after the pandemic.

**Table:** 

**What is Known:** • *Empyema is a severe complication of pneumonia.* • *Some studies have suggested increase in empyemas in Europe, but no data available for Italy.*	
**What is New:** • *The incidence of empyema in Italy significantly increased during the post-pandemic phase.* • *An increased odds of severe outcomes was observed during the COVID period.*

**Supplementary Information:**

The online version contains supplementary material available at 10.1007/s00431-025-06411-2.

## Background

Pleural empyema is a serious complication of community-acquired pneumonia (CAP) in children [1], primarily caused by infections with *Streptococcus pneumoniae*, *Streptococcus pyogenes*, and *Staphylococcus aureus* [2, 3]. Historically, concurrent or recent viral infections, such as influenza or chickenpox [4], have been associated as potential predisposing factors for pyogenic complications in children.

Since September 2022, several European countries (including France, Spain, Ireland, the Netherlands, Sweden, and the UK) have reported an unusual increase in cases of empyema and invasive group A streptococcal (iGAS) disease among children [5, 6].

This change in epidemiology has coincided with recent severe outbreaks of viral infections, including influenza, RSV, and SARS-CoV-2, following an unusual period of reduced respiratory pathogen circulation during the first 2 years of the COVID-19 pandemic [7], largely due to non-pharmacological measures such as mask mandates and school closures [8].

No formal reports have assessed the impact of the pandemic on the diagnoses, etiology, and outcomes of severe pneumonia in Italian children. Therefore, with the hypothesis that the pandemic may also have led to an increased incidence and severity of pediatric empyemas in Italy, we implemented this national multicenter study.

## Methods

### Study population

We conducted a nationwide multicenter retrospective observational study, inviting all the institutions included in the network of the Italian Society of Pediatric Infectious Diseases (SITIP). The study involved secondary and tertiary care institutions providing pediatric care across Italy. Specifically, among the 16 participating centers, 8 were pediatric departments within university or teaching hospitals, 3 were standalone children’s hospitals, and the remaining 5 were pediatric units embedded within general hospitals. Participating centers were invited to enroll all in-patient children younger than 18 years of age and diagnosed with pleural empyema between January 1, 2018, and December 31, 2023. For the study purpose, pleural empyema was defined as the presence of pus in the pleural space [9], documented by chemical and/or microbiological (direct microscopy and/or culture and/or molecular) detection in the pleural fluid, and/or radiological diagnostics (purulent material in the pleural cavity documented by ultrasound or pleural fluid documented by lung computed tomography [CT] scan), identified by ICD code. Patients were excluded if aged 18 years or older, or in case of refusal from legal representatives to consent to the use of clinical and personal data for scientific and research purposes. In addition, we excluded patients with immune deficiencies or patients meeting criteria for hospital-acquired respiratory infections.

### Variables and procedures

Data about demographic, clinical, microbiological, and radiological presentation, as well as information about surgical procedures, antibiotics, and outcomes, were collected for each patient. Data were collected using an anonymized Excel sheet shared by the investigator with participating centers, protected by a different password for each center. To investigate possible changes in the number and clinical case presentation, all patients were classified into three phases [10]: “pre-covid” phase, from January 2018 to December 2019, when the first case was described in China; “COVID” phase, from January 2020 to December 2021, during the first severe COVID-19 waves when most non-pharmacological interventions were implemented; “post-pandemic” phase, from January 2022 to December 2023, when the SARS-CoV-2 virus became endemic and social restrictions were dismissed. The incidence was calculated based on the national pediatric resident population (< 18 years) during the different phases (we used the latest available sources: https://demo.istat.it/app/?l=it&a=2019&i=POS and http://dati.istat.it/Index.aspx?QueryId=18977).

### Primary and secondary objectives

The primary aim of the study was to compare the disease severity during the three periods.

Different criteria were used to classify case severity, including clinical, laboratory, and radiological parameters:

- Clinical severity: defined by the need for oxygen therapy upon admission.
- Laboratory severity: defined by a C-reactive protein (CRP) level greater than 10 mg/dL and/or a procalcitonin (PCT) level greater than 2 ng/mL on admission.
- Radiological severity: defined by the presence of cavitation on chest X-ray or CT scan, and/or the detection of septations on lung ultrasound (LUS) or CT scan.

In addition, to better investigate possible risk factors for severe outcomes, the need for advanced respiratory support (CPAP and/or mechanical ventilation), pediatric intensive care unit (PICU) admission, video-assisted thoracic surgery (VATS) or surgical resection, and death were merged to build a composite variable (defined as “severe outcome”).

The comparisons between the three study periods were also performed for the following secondary outcomes: etiology, surgical approach (i.e., drainage, thoracoscopy, and fibrinolysis), and length of hospital stay.

In addition, we classified as “complicated effusions” those with the presence of fibrin strands in the pleural fluid on lung ultrasound or suprafluid density of pleural effusion at the CT scan.

“Respiratory distress” was defined when one or more signs of respiratory distress were present (grunting, nasal flaring, retractions, accessory muscle use). Patients fulfilling the criteria for hospital-acquired infections were excluded, and we only included patients arriving from the community with no recent admissions within the previous 90 days.

### Statistical analysis

For continuous variables, medians (IQRs) or means (SDs) were presented as appropriate based on normality assumption tested by the Shapiro–Wilk test. Categorical variables were presented as counts and percentages. Continuous outcomes were studied with the Kruskal–Wallis test or ANOVA according to distribution. A multiple logistic regression model was then built for the outcome “clinical/composite severity,” including clinically relevant variables as covariates. Goodness of fit of the model was assessed with the Hosmer–Lemeshow test. Possible final models were evaluated by comparing their respective Akaike information criterion (AIC) and Bayesian information criterion (BIC), where indicated. For the outcomes “pediatric intensive care unit (PICU) admission,” “use of high-flow nasal cannula (HFNC),” and “use of mechanical ventilation,” a mixed-effect logistic regression model was performed, which included the same clinically relevant variables as above as fixed variables and a random effect on the center. Intraclass correlation was then calculated. Missing data were analyzed and considered to be missing completely at random (MCAR) and dealt with by complete-case analysis; details are provided in the supplementary material. Data were analyzed with Stata 18.0 B.E. (StataCorp LLC, USA). Two-tailed tests were used. *P*-values < 0.05 were considered significant [11].

## Ethical approval

The study was authorized by the ethics committee of the Fondazione Policlinico Universitario A. Gemelli IRCCS of Rome, Italy (Ethics approval ID6199, Protocol no. 0035081/23) and, subsequently, by those of the other participating institutions, and performed in accordance with the Declaration of Helsinki. Informed consent was obtained from all patients or legal guardians to use their anonymized data for scientific purposes, including publication.

## Results

### Study population and disease severity

A total of 266 patients hospitalized in 19 Italian institutions were included in the study. Their age ranged from 29 days to 17 years, with a median age of 4 years (IQR 2–7). The majority (72.1%) were of Italian origin, and 163 (61.3%) were female. Forty-eight patients (18.0%) had at least one comorbidity, the most representative of which were neurological diseases (33.3%) (Table 1). Almost half of the patients (43.0%) had already received antibiotics before hospital admission, and 3 patients (1.1%) were chronically treated with immunosuppressants.

**Table 1:** Clinical, laboratory, and radiological characteristics of the study population

General characteristics	*N* = 266
Female sex, *n* (%)	103 (38.7)
Median age, years (IQR)	4.0 (2.0–7.0)
Ethinicity, *n* (%)	
Italian	188 (70.6)
Other Europen countries	29 (10.9)
African	20 (7.5)
Asian	14 (5.3)
Latino	8 (3.0)
North American	1 (0.4)
Missing	4 (1.5)
Comorbidities, *n* (%)	48 (18.0)
Underlying chronic diseases, *n* (%)	
Neurological	17 (35.4)
Chronic respiratory	6 (12.5)
Genetic	6 (12.5)
Cardiac	5 (10.4)
Rheumatological	3 (6.2)
Oncological	2 (4.2)
Immunodeficiency	1 (2.1)
Other	12 (25.0)
Immunosuppressive treatment (ongoing), *n* (%)	3 (1.1)
Antibiotic therapy before admission, *n* (%)	114 (43.0)
**Clinical and laboratory characteristics**	
Fever, *n* (%)	258 (97.4)
Fever days, median (IQR)	6.0 (3.0–10.0)
Cough, *n*/265 (%)	200 (75.5)
Respiratory distress, *n*/265 (%)	174 (65.7)
Chest pain, *n*/265 (%)	83 (31.3)
WBC measured	262
WBC (cell10^3^/μL), median (IQR)	17.2 (12.2–24.1)
Increased WBC, *n*/262 (%)	167 (63.7)
Neutrophils (cell10^3^/μL), median (IQR)	13.0 (7.9–19.0)
Increased neutrophils, *n*/262 (%)	191 (74.3)
CRP measured	266
C-reactive protein value (mg/dL), median (IQR)	20.7 (13.5–30.6)
Increased C-reactive protein, *n* (%)	263 (99.6)
PCT measured	167
Procalcitonin value (ng/mL), median (IQR)	4.9 (0.7–21.1)
Increased procalcitonin, *n*/167 (%)	131 (78.4)
**Radiological characteristics**	
Lung US done, *n* (%)	223 (83.8)
Lung US complicated effusion, *n*/227 (%)	200 (88.1)
CXR done	261
CXR consolidation, *n*/261 (%)	241 (92.3)
CXR effusion, *n*/261 (%)	248 (95.0)
CXR cavitation, *n*/261 (%)	26 (9.9)
CT scan done	210
CT consolidation, *n*/210 (%)	197 (93.8)
CT effusion, *n*/210 (%)	204 (97.1)
CT complicated effusion, *n*/210 (%)	134 (63.8)
CT cavitation, *n*/210 (%)	74 (35.2)
**Respiratory support**	227 (85.4)
Low flow oxygen, *n*/227 (%)	106 (46.7)
HFNC, *n*/227 (%)	75 (33)
CPAP, *n*/227 (%)	10 (4.4)
NIV, *n*/227 (%)	4 (1.7)
MV, *n*/227 (%)	32 (14.1)
PICU admission, *n*/263 (%)	101 (38.4)
Video assisted thoracic surgery, *n*/259 (%)	70 (27)
Fibrinolysis, *n*/258 (%)	121 (46.9)
Surgical resection, *n*/259 (%)	16 (6.2)
**Classification of severe cases**	
Clinical severity, *n*/265 (%)	142 (53.6)
Laboratory severity,* n*/265 (%)	227 (85.7)
Radiological severity, *n*/265 (%)	241 (90.9)
“Composite” severity, *n*/266 (%)	155 (58.3)

*IQR*, interquartile range; *US*, ultrasound; *CT*, computed tomography; *CXR*, chest X-ray; *HFNC*, high-flow nasal cannula; *CPAP*, continuous positive airway pressure; *NIV*, non-invasive ventilation; *MV*, mechanical ventilation; *PICU*, pediatric intensive care unit. Increased WBC and neutrophils based on “Area Pediatrica,” available at https://www.area-pediatrica.it/archivio/2881/articoli/29050/

Fever and cough were the most common signs/symptoms at presentation, respectively reported in 97.4 and 75.5% of patients. In 85.7% of children, an increase of inflammatory markers was detected, defined as a C-reactive protein (CRP) greater than 10 mg/dL and/or a procalcitonin (PCT) greater than 2 ng/mL. The clinical and laboratory characteristics at admission are described in detail in Table 1. When performed, lung ultrasound detected empyema in 88.1% of cases. In 92.7% of cases, a lung consolidation was visible on the chest x-ray, whereas cavitation was found only in 10.0% of cases on X-ray and in 35.2% of cases on CT scan. Radiological findings are reported in detail in Table 1.

As regards the overall disease severity, 142 children (53.6%) satisfied the criteria for clinical severity, 227 children (85.7%) for laboratory severity, and 241 (90.9%) for radiographic severity. No fatality was reported, but 101 (38.4%) required intensive care assistance. Two hundred three patients (87.9%) required a surgical procedure.

### Comparison between the three study periods

The comparison in terms of clinical characteristics, etiology, therapeutic approach, and outcome across the three different phases was performed for 265 patients, with one patient excluded due to recurrent empyema, which made categorization challenging.

**Fig. 1:**
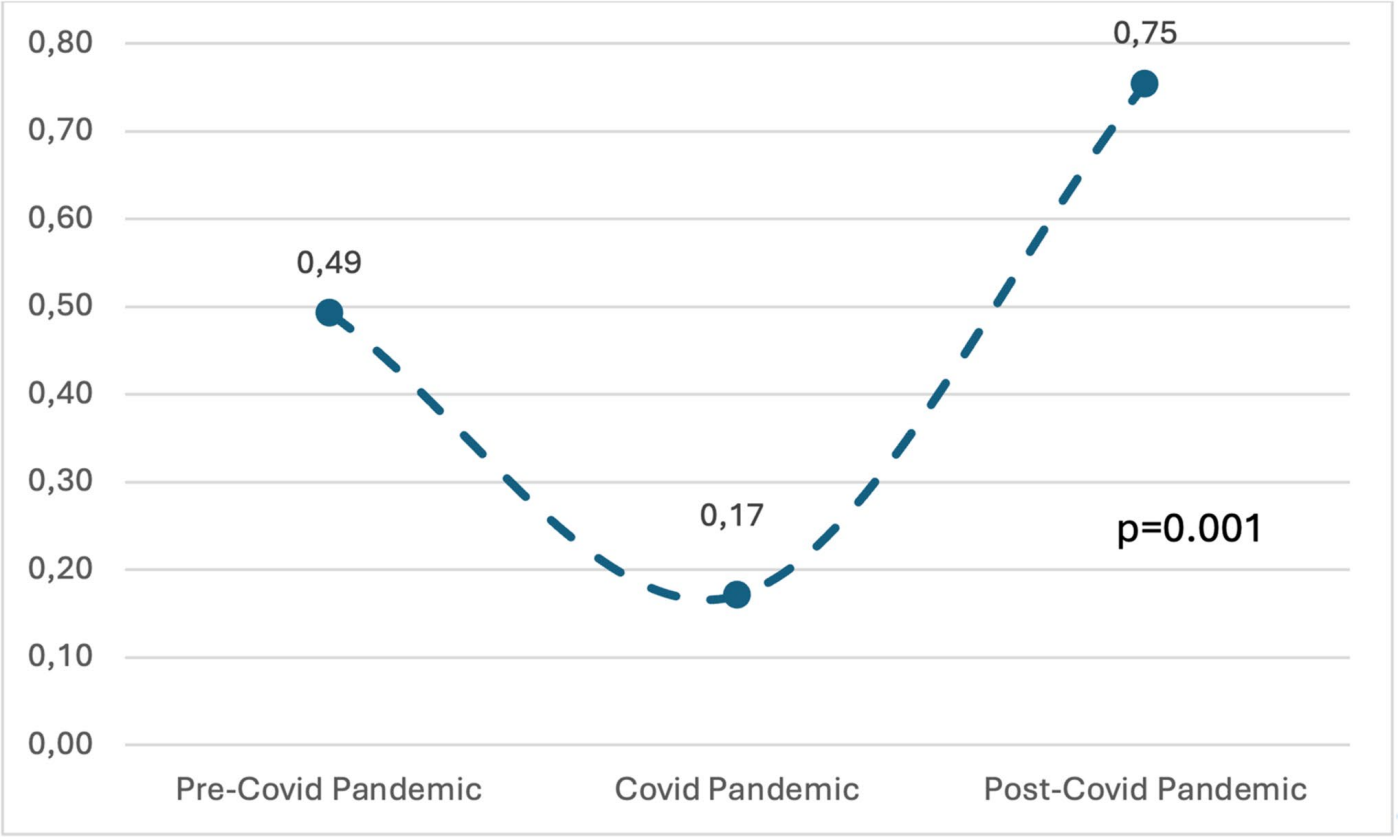
Incidence of empyema per 100,000 pediatric inhabitants during the three study periods of observation

Of 265 total cases of empyema, no differences in the demographic characteristics (age, sex, and ethnicity) between the three phases were observed. Clinical characteristics, etiology, therapeutic approach, and outcomes are summarized in Table 2. The frequency of empyema was significantly different in the three phases: 95 (35.8%) were reported during the pre-COVID phase, 32 (12.1%) during the COVID phase, and 138 (52.1%) during the post-pandemic phase (*p* = 0.001). The incidence of empyema significantly increased during the post-pandemic phase (pre-COVID 95/19,288,639 [0.49], during COVID pandemic 32/18,784,272 [0.17], post-pandemic 138/18,294,627 [0.75], *p* = 0.001) (Fig. 1).

**Table 2:** Etiology, therapeutic approach, and clinical outcome of children with pleural empyema according to period of observation

	Pre-COVID	COVID pandemic	Post-COVID	
General characteristics, *n* (%*)	*N* = 95	*N* = 32	*N* = 138	*P*
Age, months, median (IQR)	49.0 (32.0–89.0)	50.5 (23.5–114.0)	51.0 (30.0–84.0)	0.91
Comorbidities	17 (17.9)	9 (28.1)	22 (15.9)	0.27
Respiratory distress	54 (57.4)	21 (65.6)	98 (71.0)	0.10
Antibiotic therapy before admission	43 (45.7)	9 (28.1)	62 (44.9)	0.18
Fever, days	7.0 (4.0–10.0)	6.0 (3.0–10.0)	6.0 (3.0–10.0)	0.6
Cough	62 (66.0)	22 (68.8)	116 (84.1)	0.004
Respiratory distress	54 (57.4)	21 (65.6)	98 (71.0)	0.1
Chest pain	30 (31.9)	11 (34.4)	41 (29.7)	0.85
Etiology, *n* (%)				
Bacterial infections				
*Staphylococcus aureus*	1 (1.1)	6 (18.8)	5 (3.6)	< 0.001
*Streptococcus pneumoniae*	15 (15.8)	4 (12.5)	34 (24.6)	0.13
*Streptococcus pyogenes*	5 (5.3)	0 (0.0)	15 (10.9)	0.064
*Mycoplasma pneumoniae*	3 (3.2)	0 (0.0)	0 (0.0)	0.066
*Klebsiella pneumoniae*	0 (0.0)	1 (3.1)	0 (0.0)	0.026
*Haemophilus influenzae*	3 (3.2)	2 (6.2)	7 (5.1)	0.7
Viral infection	3 (3.2)	0 (0.0)	6 (4.3)	0.47
Viral co-infection	1 (1.1)	0 (0.0)	6 (4.3)	0.19
Polimicrobial	4 (4.2)	1 (3.1)	11 (8.0)	0.38
Treatment				
Total antibiotic treatment, median length in days (IQR)	26.0 (17.0–40.0)	28.0 (22.0–40.0)	28.0 (19.0–38.0)	0.74
Intravenous antibiotic treatment median length in days (IQR)	20.0 (12.0–28.0)	21.0 (16.5–29.5)	19.0 (14.0–28.0)	0.33
Pleural drainage	72 (78.3)	30 (93.8)	129 (94.2)	< 0.001
Fibrinolysis	36 (40.4)	21 (67.7)	63 (46.0)	0.031
Visual assisted thoracic surgery	23 (25.6)	9 (29.0)	38 (27.7)	0.91
Surgical resection	4 (4.4)	4 (12.9)	8 (5.8)	0.23
Clinical outcome				
Clinical severity	38 (40.4)	21 (65.6)	82 (59.4)	0.006
Laboratory severity	73 (77.7)	29 (90.6)	125 (90.6)	0.015
Radiological severity	84 (89.4)	28 (87.5)	128 (92.8)	0.52
Severe outcome (composite)	47 (49.5)	24 (75.0)	84 (60.9)	0.029
PICU	31 (32.6)	15 (46.9)	55 (39.9)	0.296
Oxygen therapy	50 (53.8)	29 (90.6)	109 (79.6)	< 0.001
Respiratory support				
Oxygen low flow	43 (45.2)	12 (37.5)	50 (36.2)	0.370
HFNC	11 (11.5)	15 (46.8)	49 (35.5)	< 0.001
CPAP	3 (3.1)	0 (0.0)	7 (5.1)	0.368
NIV	1 (1.0)	0 (0.0)	3 (2.4)	0.596
MV	11 (1.5)	4 (12.5)	17 (12.3)	0.982
Length of stay, median in days (IQR)	22.0 (13.0–31.0)	25.0 (18.0–32.0)	21.0 (16.0–29.0)	0.23

*Calculated based on available data. *IQR*, interquartile range; *PICU*, pediatric intensive care unit; *HFNC*, high flow nasal cannula; *CPAP*, continuous positive airway pressure; *NIV*, non-invasive ventilation; *MV*, mechanical ventilation

Overall, *S. pneumoniae, S. pyogenes*, and *S. aureus* were the commonest isolated pathogens. The types of microbiological isolates were similar across the time periods (Table 2), except for an increase in *S. aureus* detection during the COVID pandemic. Blood culture and pleural fluid examinations became more frequent, as well as molecular assays (polymerase chain reaction), during the COVID and post-pandemic phases, although differences were not statistically significant (Table S1).

No differences were detected either in the total duration of antibiotic therapy—oral plus intravenous—or intravenous alone.

A significant increase in the use of pleural drainage was observed between the pre-COVID phase and the subsequent phases.

**Fig. 2:**
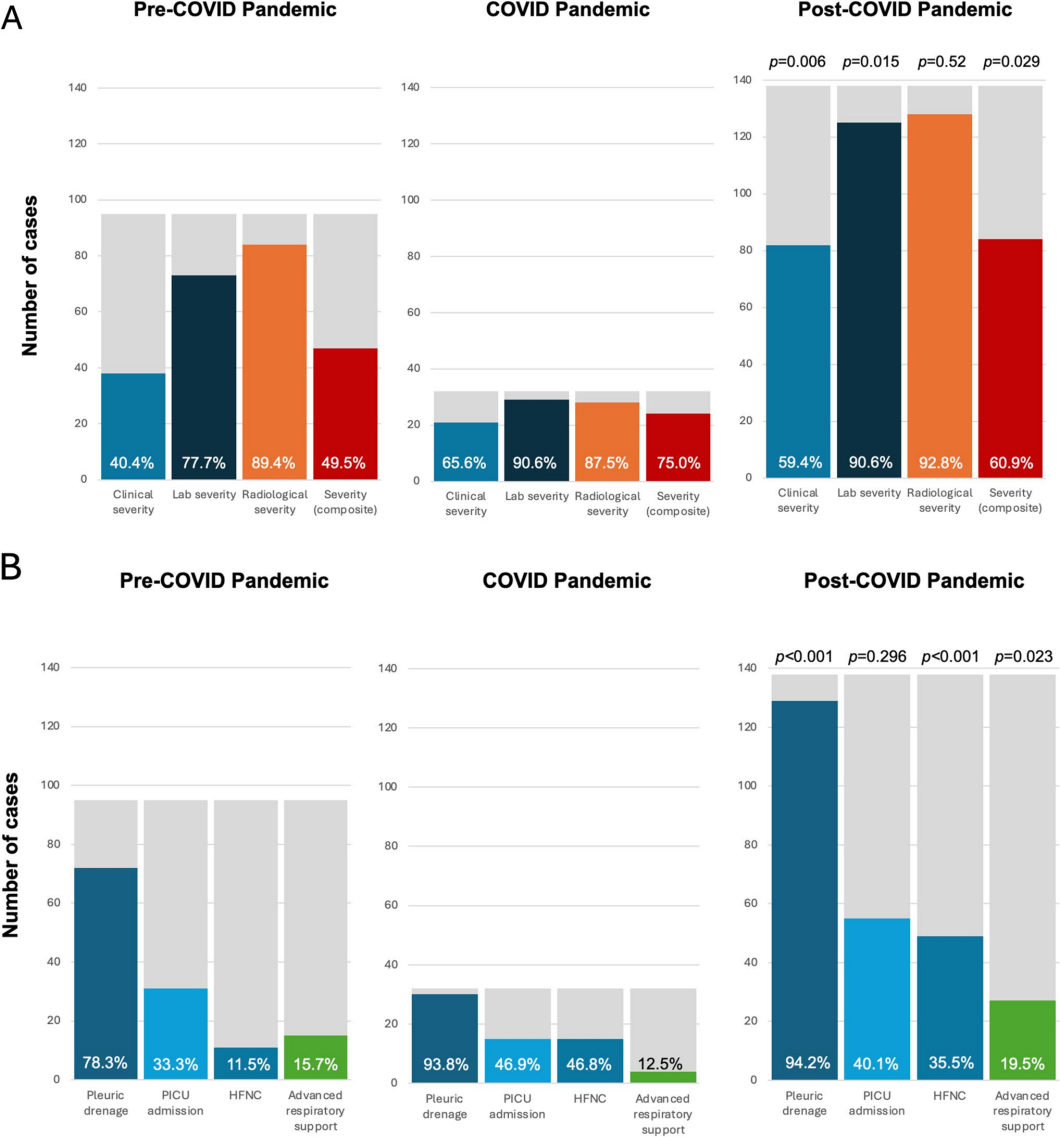
Differences in disease severity during the three study periods of observation. Figure legend: **A** The total number of patients for each time period (gray) and the relative percentage (full colors) of clinical, laboratory, and radiological severity observed during the three periods of observation. **B** The total number of patients for each time period (gray) and relative percentage (full colors) in the need of invasive therapeutic approaches applied during the three study periods, including the need for pleural drainage, pediatric intensive care unit admission, and advanced respiratory support. *P*-values reflect the results of statistical comparison (chi-squared test) between the frequency of each outcome during the pre-COVID pandemic and the COVID and post-COVID pandemic periods. *PICU*, pediatric intensive care unit; *HFNC*, high-flow nasal cannula; advanced respiratory support includes children receiving *CPAP*, continuous positive airway pressure*; NIV,* non-invasive ventilation; or *MV,* mechanical ventilation

In the COVID and post-pandemic phases, a significant increase in clinical and laboratory severity was observed (*p* = 0.006; *p* = 0.015), while no significant differences were noted in radiological severity (Fig. 2A). Severe composite outcome was also more frequent in the COVID and post-pandemic phases compared to the pre-pandemic period (*p* = 0.029) (Table 3).

**Table 3:** Factors affecting the risk of severe outcome, PICU admission, and use of HFNC and MV

	Severe outcome				PICU admission				HFNC				Mechanical ventilation			
Factors	OR	95%CI		*P*	OR	95%CI		*P*	OR	95%CI		*P*	OR	95%CI		*P*
Period of observation																
Pre-COVID period	ref	-	-		Ref	-	-		ref	-	-		ref	-	-	
COVID pandemic	3.42	1.21	9.65	**0.020**	1.53	0.53	4.43	0.427	12.47	3.25	47.74	** < 0.001**	1.04	0.14	7.33	0.963
Post-COVID period	1.54	0.79	2.99	0.204	1.35	0.63	2.89	0.437	4.71	1.64	13.54	**0.004**	1.66	0.50	5.51	0.403
**Patients’ and clinical characteristics**																
Female	1.47	0.78	2.77	0.232	1.22	0.62	2.40	0.551	1.04	0.46	2.33	0.911	3.53	1.12	11.04	**0.031**
Age	0.99	0.98	1.00	0.169	0.98	0.98	0.99	**0.002**	0.99	0.99	1.00	0.996	0.97	0.97	0.99	**0.012**
Comorbidity	2.18	0.82	5.77	0.114	3.46	1.23	9.70	**0.018**	1.56	0.48	5.05	0.451	7.97	1.66	39.22	**0.009**
Length of fever	1.10	1.03	1.18	**0.004**	1.09	1.01	1.17	**0.025**	1.07	0.97	1.18	0.135	1.05	0.93	1.19	0.420
Elevated WBC	0.60	0.25	1.43	0.252	1.12	0.43	2.94	0.809	0.16	0.05	0.51	**0.002**	6.32	0.87	45.71	0.068
Elevated neutrophiles	2.02	0.78	5.24	0.147	1.28	0.44	3.75	0.644	7.74	1.97	30.26	**0.003**	0.35	0.05	2.52	0.298
Elevated CRP	1.00	0.97	1.02	0.950	1.00	0.98	1.02	0.645	0.99	0.96	1.02	0.827	0.99	0.95	1.04	0.785
Antibiotics before admission	1.06	0.57	1.95	0.846	0.67	0.34	1.31	0.251	1.44	0.65	3.21	0.361	0.55	0.16	1.84	0.338
Complicated effusion at lung US	3.29	1.26	8.57	**0.015**	3.01	0.90	10.08	0.073	3.88	0.78	19.25	0.096	0.83	0.15	4.56	0.831
					ICC 0.08				ICC 0.31				ICC 0.49			

Results of logistic regression (for the outcome “clinical severity”) and mixed-effects logistic regression for the outcomes “PICU admission,” “use of HFNC,” and “use of MV.” Length of fever refers to the duration of a body temperature higher than 38 °C, expressed in days. *OR*, odds ratio; *HFNC*, high flow nasal cannula; *MV*, mechanical ventilation; *CRP*, C-reactive protein; *ICC*, intra-class correlation. Increased WBC and neutrophils based on “Area Pediatrica,” available at https://www.area-pediatrica.it/archivio/2881/articoli/29050/

The need for HFNC and advanced respiratory support [defined as continuous positive airway pressure (CPAP), non-invasive ventilation (NIV), or mechanical ventilation (MV)] was significantly more frequent in the COVID and post-pandemic phases (*p* < 0.001; *p* = 0.023), while no difference in the need for PICU admission was observed (Fig. 2B).

We have incomplete data on the use of NSAIDS before admission, as some centers did not report it (89 answers; 79/89 used and 10/89 did not). While we acknowledge this constitutes a limitation, we describe a stable use of them across the three periods (chi^2^ *p* = 0.67).

Use of paracetamol before admission did not show significant changes over time.

An increased odds of severe outcomes was observed during the COVID period (OR: 3.428, 95% CI: 1.21–9.65, *p* = 0.020) and in patients with complicated effusion observed at lung ultrasound (OR: 3.29, 95% CI: 1.26–8.57, *p* = 0.015). Each day of persistent fever was associated with a 10% increased risk of severe outcome (OR: 1.10, 95% CI: 1.03–1.18, *p* = 0.004).

In our study population, the occurrence of severe outcome was not associated with the etiology of empyema. According to logistic regression analysis, the identification of the most isolated pathogens, either *Staphylococcus aureus*, *Streptococcus pneumoniae*, or *S. pyogenes*, was not associated with an increased chance of severe outcome (data not shown).

## Discussion

Our six-year nationwide multicenter retrospective study provides a detailed analysis of the diagnostic pathway and clinical outcomes of pleural empyema before and after the COVID-19 pandemic in a large pediatric population living in Italy.

Pediatric pleural empyema usually results from bacterial pneumonia and occurs more frequently in young children, especially between 2 and 4 years [12], in keeping with the findings in our population. Some underlying conditions may increase the risk of pleural empyema in children; in our study population, About 20% had an associated condition, with neurological diseases being the most common type of comorbidity.

Our primary aim was to investigate possible changes in the incidence and severity of empyema in children admitted before, during, and after the COVID-19 pandemic. The analysis of our study population, collected by 19 participating institutions in Italy, documented a significant increase in the absolute number of empyema cases per year in the post-pandemic period, consistent with findings from studies on other bacterial infections [5, 13–16]. The increase resulted in a 50% rise in the incidence rate compared to the pre-COVID period. The surge in empyema cases during the post-pandemic period was accompanied by a significant increase in the number of cases with severe clinical and laboratory presentations, with increased need for oxygen or HFNC, pleural drainage, and PICU admission.

The increased incidence and severity of bacterial infection after the COVID-19 pandemic was initially attributed to the so-called immunity debt, due to the previous use of non-pharmaceutical interventions (mask wearing, hand hygiene, social distancing, travel restrictions, and school closures) [17]. Restrictions applied during the pandemic limited viral circulation and, consequently, bacterial superimposed infections. They also reduced exposure to some specific bacteria such as S. pneumoniae and group A *Streptococcus*. Moreover, during the pandemic, a delay in vaccination programs was reported, with a substantial reduction of pneumococcal coverage [18]. In our population, we observed only minor changes in the microbial isolates from blood, pleural, and bronchoalveolar samples collected during the three study periods.

It is likely that not only non-pharmaceutical interventions, but also additional factors, may have contributed to the increased bacterial infection rate observed in the months following the COVID pandemic. A recent study suggests a more complex role of SARS-CoV-2 infection, which seems to cause a long-term reduction of innate and adaptive immune cells, including granulocytes, monocytes, and lymphocytes [19, 20].

We observed a significant increase in the incidence of severe empyema cases in children, as evidenced by the increased need for oxygen and HFNC therapy during and even after the pandemic period. This was accompanied by a rising number of children presenting with elevated inflammatory markers or requiring pleural drainage, as well as an overall increase in the rate of children experiencing a composite severe outcome. The mixed-effects logistic regression analysis revealed that, beyond the timing of presentation, other host-related factors influenced the clinical outcomes of children with empyema. Specifically, older age and the presence of underlying comorbidities were associated with a higher likelihood of PICU admission and the need for mechanical ventilation. Likewise, the presence of prolonged fever increased the odds of a severe outcome and the need for intensive care by 10%.

As a secondary aim, we investigated changes in the diagnostic approach to these children during the study period. Initial evaluation of a child with suspected parapneumonic empyema is generally based on chest radiography; yet, thorax ultrasonography is the preferred method to determine the size of the effusion, describe its nature more accurately, and identify the presence of septations and loculation, thus supporting the choice between conservative and invasive treatment [21]. Indeed, ultrasonography was reported in 84% of our patients and detected empyema in 88% of cases. Although not statistically significant, a slight increase in the use of ultrasonography was observed in the post-pandemic period. This was probably based on a huge increase in the use of lung ultrasound for COVID-19 patients, both in adults and children [22, 23]. Based on our results, the evidence of a complicated effusion observed during lung US was associated with a threefold increased chance of severe composite outcome. This evidence further supports the role of lung ultrasonography in the management of these children, as a simple, easily reproducible, and non-invasive diagnostic tool. On the other hand, CT imaging should only be considered in refractory cases to rule out other causes for pleural effusion or complications like lung necrosis or abscess [24]. Although not generally recommended, in our population CT was performed in almost 80% of children, but led to the detection of a cavitary lung lesion only in one-third of these cases. Thus, the initial choice of CT imaging in a child with empyema should be better revised.

As a legacy of the COVID experience, we have learned to use better and more frequent microbiological tests for screening of respiratory pathogens, on blood or pleural effusion. Due to technical advances, the rate of positive molecular assays became higher, with a significant increase in the isolation of *S. aureus* from either blood or bronchoalveolar lavage samples during the COVID pandemic (Supplementary data). Geslain et al. reported 92% sensitivity and 95% specificity of the multiplex PCR assay on bronchial samples in critically ill pediatric patients [25, 26]. Although culturing with antimicrobial susceptibility testing remains the reference method, rapid-multiplex PCR assays may lead to early appropriate therapeutic choice, including use and selection of antibiotic therapy, resulting in more appropriate treatment. Some of the extra costs of the pandemic era resulted in major investments in diagnostic technologies, especially in microbiology, which now give us their payback in terms of expertise and equipment for the care of adults and children with respiratory disorders.

Interestingly, an increased use of procalcitonin was observed in the pandemic and post-pandemic periods.

The main limitation of our study is its retrospective design. Additionally, the lack of investigation into viral co-infections represents another limitation, as upper respiratory tract viral infections may predispose children to the development of severe pneumonia, in particular considering the impact of the pandemic on the burden of respiratory viruses [27, 28] and how this impacted the changed landscape of infections in general [29, 30]. A further limitation is the unavailability of precise data regarding the number of patients accessing each individual institution, including the number of pneumonia cases in Italy each year. However, we estimated the incidence of empyema by using data from the local population under 18 years of age during each study period. Given the consistency of the data from the national statistical institute, we are confident that this extrapolation reliably reflects the increase in empyema diagnoses across the Italian regions. We have not included other pertinent outcomes like the presence of concurrent septic shock or acute kidney injury. These may be good markers of the severity of the illness as well. Also, key severity biomarkers like PCT were not uniformly available, and this may have influenced severity classification or regression findings.

Finally, the limited data on the use of non-steroidal anti-inflammatory drugs prior to admission in our population precluded any statistical analysis, despite this being a relevant risk factor based on current evidence.

However, the large sample size and the nationwide distribution of the participating institutions from the North, Center, and South of Italy represent a major strength of the study. To date, no Italian study and only a limited number of European studies have provided adequately representative national data on the surge in empyema following the COVID-19 pandemic.

In conclusion, our 6-year observational study confirmed an increase in both the incidence and severity of empyema in children accessing Italian healthcare institutions. This trend may be explained by multiple factors, including the impact of SARS-CoV-2 transmission and the effects of related preventive measures on epidemiology, also affecting, in the long run, the child immune systems. Age and the presence of comorbidities were the key determinants for the need for mechanical ventilation and intensive care. All cases were successfully managed with no fatalities; however, an increased demand for high-flow nasal cannula (HFNC) therapy was observed during and after the pandemic.

## Supplementary Information

Below is the link to the electronic supplementary material.

Supplementary file1 (DOCX 400 KB)

## Data Availability

No datasets were generated or analysed during the current study.

## Declarations

The study was authorized by the ethics committee of the Fondazione Policlinico Universitario A. Gemelli IRCCS of Rome, Italy (Ethic approval ID6199, Protocol no. 0035081/23) and, subsequently, by those of the other participating institutions, and performed in accordance with the Declaration of Helsinki. Informed consent was obtained from all patients or legal guardians to use their anonymized data for scientific purposes including publication.
