# Correction to: Incidence, etiologies, and outcomes of severe pediatric community-acquired empyema before and after the pandemic: an Italian multicentric study

**DOI:** 10.1007/s00431-025-06521-x

**Published:** 2025-10-24

**Authors:** Danilo Buonsenso, Carlotta Montagnani, Lorenza Romani, Anna Camporesi, Lucia Scarlato, Marco Denina, Daniele Zama, Aldo Naselli, Sonja Montonesi, Ilaria Liguoro, Marcello Mariani, Fabio Cardinale, Elena Chiappini, Maia De Luca, Silvia Garazzino, Samantha Bosis, Giulia Pattarino, Valentina Burzio, Luca Di Napoli, Claudia Colomba, Giangiacomo Nicolini, Giulia Lorenzetti, Luisa Galli, Andrea Lo Vecchio, SITIP Empyema Working Group

**Affiliations:** 1 Pediatric Infectious Diseases, Pediatric Emergency Care and Pediatric Ultrasound Department of Woman and Child Health and Public Health and Fondazione, Policlinico Universitario “A. Gemelli,”, Rome, Italy; 2 Pediatric Infectious Diseases Unit, Meyer Children’s Hospital IRCCS, Florence, Italy; 3 Infectious Diseases Unit, Bambino Gesù Children’s Hospital, IRCCS, Rome, Italy; 4 Infectious Diseases Unit, Department of Pediatrics, University of Turin, Regina Margherita Children’s Hospital, Turin, Italy; 5 Department of Medical and Surgical Sciences, Alma Mater Studiorum, University of Bologna, Bologna, Italy; 6 Pediatric Emergency Unit, IRCCS Azienda Ospedaliero-Universitaria Di Bologna, Bologna, Italy; 7 Neonatal Intensive Care Unit, Transmural Pediatric Department, S. Chiara Hospital, Trento, Italy; 8 Department of Pediatrics, Hospital of Bolzano, Bolzano, Italy; 9 Division of Pediatrics, University Hospital of Udine, Udine, Italy; 10 Pediatric Infectious Diseases Unit—IRCCS Istituto Giannina Gaslini, Genoa, Italy; 11 Department of Pediatrics, Pediatric Hospital Giovanni XXIII, University of Bari, Bari, Italy; 12 Department of Health Sciences, University of Florence, Florence, Italy; 13 Fondazione IRCCS Ca’ Granda Ospedale Maggiore Policlinico, Milan, Italy; 14 Department of Mother and Child Health, Division of Pediatrics, ASST Grande Ospedale Metropolitano Niguarda, Milan, Italy; 15 Department of Health Sciences, Division of Pediatrics, University of Piemonte Orientale, Novara, Italy; 16 Arcispedale Sant’Anna, University Hospital of Ferrara, Ferrara, Italy; 17 Department of Health Promotion, Mother and Child Care, Internal Medicine and Medical Specialties, University of Palermo, Palermo, Italy; 18 Pediatric and Neonatal Pathology Unit, Medical Area Department, S. Martino Hospital, Belluno, Italy; 19 Department of Translational Medical Sciences, University of Naples Federico II, Naples, Italy; 20 S. Spirito Hospital, Azienda Sanitaria Locale, Pediatrics Pescara, Italy; 21 Azienda Ospedaliero Universitaria Policlinico Di Modena, Modena, Italy; 22 Department of Pediatrics, University of Chieti-Pescara, Chieti, Italy; 23 Pediatria E Neonatologia, Ospedale Veris Delli Ponti Di Scorrano, ASL Lecce, Lecce, Italy; 24 NEUROFARBA Department, University of Florence, Florence, Italy; 25 Pediatric Unit, Meyer Children’s Hospital IRCCS, Florence, Italy; 26 Pediatric Emergency Department, Regina Margherita Children’ S Hospital, Turin, Italy; 27 Area Pediatrica, Dipartimento Di Scienze Della Vita E Sanità Pubblica, Università Cattolica del Sacro Cuore, Rome, Italy; 28 Department of Pediatric Anesthesia and Intensive Care, Buzzi Children’s Hospital, Milan, Italy

**Correction to: European Journal of Pediatrics (2025) 184:594**

10.1007/s00431-025-06411-2

The authors regret to inform that an error occurred in the presentation of the author names in the published version of the article. Specifically, all authors' first names and surnames were inadvertently switched.

The author list should be corrected as follows:

**Published (Incorrect) Author List:**

Buonsenso Danilo^1,27^ · Montagnani Carlotta^2^ · Romani Lorenza^3^ · Camporesi Anna^1^ · Scarlato Lucia^1^ · Denina Marco^4^ · Zama Daniele^5,6^ · Naselli Aldo^7^ · Montonesi Sonja^8^ · Liguoro Ilaria^9^ · Mariani Marcello^10^ · Cardinale Fabio^11^ · Chiappini Elena^2,12^ · De Luca Maia^3^ · Garazzino Silvia^4^ · Bosis Samantha^13^ · Pattarino Giulia^14^ · Burzio Valentina^15^ · Di Napoli Luca^16^ · Colomba Claudia^17^ · Nicolini Giangiacomo^18^ · Lorenzetti Giulia^3^ · Galli Luisa^2,12^ · Lo Vecchio Andrea^19^ · SITIP Empyema Working Group^1,3,5,6,11,15,17,20,21,22,23,24,25,26^

**Corrected Author List:**

Danilo Buonsenso^1,27^ · Carlotta Montagnani^2^ · Lorenza Romani^3^ · Anna Camporesi^1^ · Lucia Scarlato^1^ · Marco Denina^4^ · Daniele Zama^5,6^ · Aldo Naselli^7^ · Sonja Montonesi^8^ · Ilaria Liguoro^9^ · Marcello Mariani^10^ · Fabio Cardinale^11^ · Elena Chiappini^2,12^ · Maia De Luca^3^ · Silvia Garazzino^4^ · Samantha Bosis^13^ · Giulia Pattarino^14^ · Valentina Burzio^15^ · Luca Di Napoli^16^ · Claudia Colomba^17^ · Giangiacomo Nicolini^18^ · Giulia Lorenzetti^3^ · Luisa Galli^2,12^ · Andrea Lo Vecchio^19^ · SITIP Empyema Working Group^1,3,5,6,11,15,17,20,21,22,23,24,25,26^

The publisher and authors sincerely apologize for this error and any inconvenience it may have caused.

The original article has been corrected.

