# Supplementary material for: Incidence, etiologies, and outcomes of severe pediatric community-acquired empyema before and after the pandemic: an Italian multicentric study
Source: Eur J Pediatr. 2025 Sep 4;184(9):594. doi: 10.1007/s00431-025-06411-2 (PMC12408734; doi:10.1007/s00431-025-06411-2)
Supplement: Supplementary file 1 — Supplementary file1 (DOCX 400 KB) [file 431_2025_6411_MOESM1_ESM.docx]

**Supplementary material**

**Table S1. Microbiological isolates in different biological samples**

| **Microbiological examinations** | **Pre-COVID**  **(n=95)** | **COVID**  **(n=32)** | **Post-COVID**  **(n=138)** |  |
| --- | --- | --- | --- | --- |
| **Blood colture performed** | **75 (78.9%)** | **27 (84.4%)** | **125 (90.6%)** | **0.044** |
| *S. pneumoniae* | 2(2.6%) | 0 | 2(1.6%) | 0.617 |
| *S. aureus* | 0 | 2 (7.4%) | 0 | 0.001 |
| *S. pyogenes* | 0 | 0 | 1 (0.8%) | 0.663 |
| *Candida spp.* | 1(1.3%) | 0 | 1(0.8%) | 0.808 |
| **Molecular blood test performed** | **27 (28.4%)** | **9 (28.1%)** | **44 (31.9%)** | **0.821** |
| Molecular blood test result (Microrganism) |  |  |  |  |
| *M. pneumoniae* | 3 (11.1%) | 0 (0.0%) | 0 (0.0%) | 0.046 |
| *S. pneumoniae* | 3 (11.1) | 2 (22.2%) | 9 (20.4%) | 0.557 |
| *S. aureus* | 0 (0.0%) | 1 (11.1%) | 0 (0.0%) | 0.018 |
| *H. influenzae* | 0 (0.0%) | 1 (11.1%) | 1 (2.3%) | 0.179 |
| *S. pyogenes* | 0 (0.0%) | 0 (0.0%) | 3 (6.8%) | 0.279 |
| *P. aeruginosa* | 0 (0.0%) | 0 (0.0%) | 1 (2.3%) | 0.660 |
| Pleural effusion microbiologic test done | 68 (73.1%) | 30 (93.8%) | 125 (90.6%) | <0.001 |
| **Pleural effusion coltural performed** | **65 (94.2%)** | **30 (100.0%)** | **119 (93.0%)** | **0.478** |
| Pleural effusion microrganism in coltural |  |  |  |  |
| *S. pnuemoniae* | 5 (7.7%) | 0 (0.0%) | 9 (7.6%) | 0.294 |
| *S. aureus* | 1 (1.5%) | 5 (16.7%) | 3 (2.5%) | <0.001 |
| *S. pyogenes* | 2 (3.1%) | 0 (0.0%) | 10 (8.4%) | 0.115 |
| *P. aeruginosa* | 0 (0.0%) | 0 (0.0%) | 1 (0.8%) | 0.669 |
| *S. anginosus group* | 2 (3.1%) | 1 (3.3%) | 1 (0.8%) | 0.459 |
| *S. maltofilia* | 0 (0.0%) | 0 (0.0%) | 1 (0.8%) | 0.669 |
| *E. coli* | 1 ( 1.5%) | 0 ( 0.0%) | 0 ( 0.0%) | 0.316 |
| *K. pneumoniae* | 0 ( 0.0%) | 1 ( 3.3%) | 0 ( 0.0%) | 0.045 |
| *F. necrophorum* | 0 ( 0.0%) | 0 ( 0.0%) | 1 ( 0.8%) | 0.669 |
| *S. marcescens* | 1 ( 1.5%) | 0 ( 0.0%) | 0 ( 0.0%) | 0.316 |
| *E. corrodens* | 0 ( 0.0%) | 0 ( 0.0%) | 1 ( 0.8%) | 0.669 |
| *H. influenzae* | 0 ( 0.0%) | 0 ( 0.0%) | 1 ( 0.8%) | 0.669 |
| **Pleural effusion molecular test performed** | **26 (27.3%)** | **10 (32.3%)** | **52 (37.6%)** | **0.251** |
| Pleural effusion molecular tests results |  |  |  |  |
| *S. pneumoniae* | 4 ( 4.2%) | 3 ( 9.4%) | 20 (14.5%) | 0.044 |
| *S. aureus* | 0 (0.0%) | 0 (0.0%) | 3 (3.8%) | 0.341 |
| *H. influenzae* | 0 (0.0%) | 1 (10.0%) | 2 (3.8%) | 0.321 |
| *S. pyogenes* | 3 (11.5%) | 0 (0.0%) | 8 (15.4%) | 0.397 |
| *P. aeruginosa* | 0 (0.0%) | 0 (0.0%) | 2 (3.8%) | 0.492 |
| *S. anginosus group* | 0 (0.0%) | 3 (30.0%) | 0 (0.0%) | <0.001 |
| *F. necrophorum* | 1 (3.8%) | 0 (0.0%) | 1 (1.9%) | 0.759 |
| **BAL coltural performed** | **25 (26.6%)** | **10 (31.2%)** | **42 (30.4%)** | **0.760** |
| BAL coltural microrganism |  |  |  |  |
| *S. pneumoniae* | 1 (4.0%) | 0 (0.0%) | 3 (7.1%) | 0.623 |
| *S. aureus* | 0 (0.0%) | 1 (10.0%) | 0 (0.0%) | 0.033 |
| *H. influenzae* | 2 (28.6%) | 0 ( 0.0%) | 1 (6.7%) | 0.409 |
| *S. pyogenes* | 0 (0.0%) | 0 (0.0%) | 1 (2.3%) | 0.655 |
| *P. aeruginosa* | 1 (4.0%) | 0 ( 0.0%) | 2 (4.7%) | 0.617 |
| *S. maltophilia* | 1 (14.3%) | 0 ( 0.0%) | 1 ( 6.7%) | 0.786 |
| *K. aerogenes* | 0 ( 0.0%) | 0 ( 0.0%) | 1 ( 6.7%) | 0.655 |
| *A.guillouiae* | 0 ( 0.0%) | 0 ( 0.0%) | 1 ( 6.7%) | 0.655 |
| *A. baumanii* | 0 ( 0.0%) | 0 ( 0.0%) | 1 ( 6.7%) | 0.655 |
| *E. cloacae* | 1 (14.3%) | 0 ( 0.0%) | 0 ( 0.0%) | 0.348 |
| **BAL molecular test performed** | **9 ( 9.8%)** | **6 (18.8%)** | **22 (16.3%)** | **0.265** |
| BAL molecular microrganism |  |  |  |  |
| *S. pnuemoniae* | 1 ( 1.1%) | 2 ( 6.2%) | 6 ( 4.3%) | 0.543 |
| *S. aureus* | 0 ( 0.0%) | 1 ( 3.1%) | 0 ( 0.0%) | 0.026 |
| *H. influenzae* | 1 (1.1%) | 2 (6.2%) | 4 ( 2.9%) | 0.555 |
| *B. pertussis* | 0 ( 0.0%) | 0 ( 0.0%) | 1 ( 0.7%) | 0.704 |
| *K. aerogenes* | 0 ( 0.0%) | 0 ( 0.0%) | 1 ( 0.7%) | 0.704 |
| *S. pyogenes* | 0 ( 0.0%) | 0 ( 0.0%) | 2 ( 1.4%) | 0.486 |
| *M. catharralis* | 0 ( 0.0%) | 0 ( 0.0%) | 2 (1.4%) | 0.486 |
| *P. aeruginosa* | 0 ( 0.0%) | 0 ( 0.0%) | 1 ( 0.7%) | 0.704 |

Supplemantary figure S1. Microbiological isolates


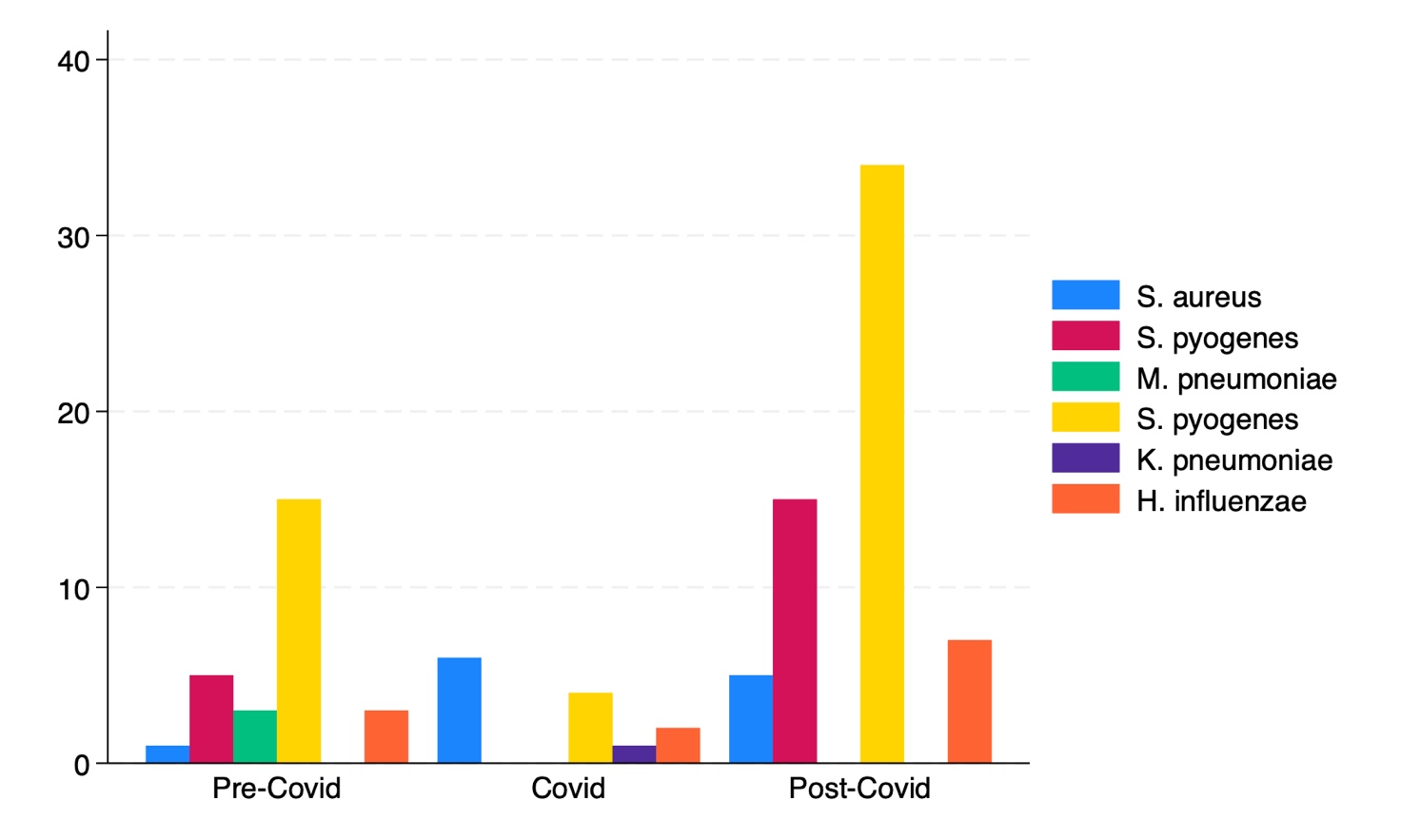


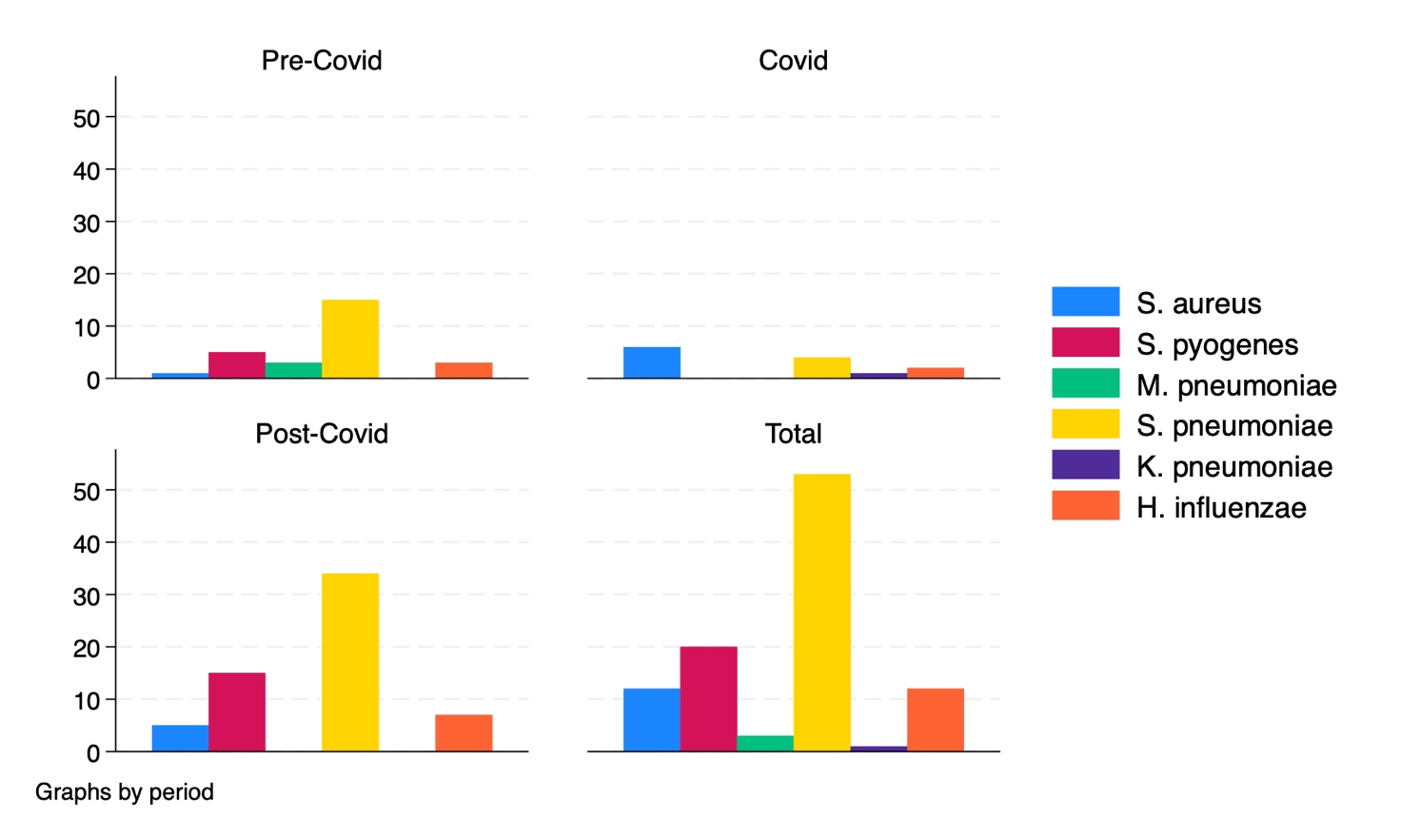


**Table S2. Distribution of first-line antibiotic treatment according to study periods**

|  |  | Pre-Covid | Covid | Post-Covid | p-value |
| --- | --- | --- | --- | --- | --- |
|  |  | N=95 | N=32 | N=138 |  |
| Antibiotic Therapy |  | 94 (98.9%) | 32 (100.0%) | 137 (99.3%) | 0.84 |
|  | Missing | 1 ( 1.1%) | 0 ( 0.0%) | 1 ( 0.7%) |  |
| Name of 1st atb prescribed | Ceftriaxone | 55 (57.9%) | 22 (68.8%) | 77 (55.8%) | 0.19 |
|  | Pip/Tazo | 5 ( 5.3%) | 4 (12.5%) | 13 ( 9.4%) |  |
|  | Amoxi/Clav | 11 (11.6%) | 1 ( 3.1%) | 6 ( 4.3%) |  |
|  | Vancomicin | 4 ( 4.2%) | 1 ( 3.1%) | 4 ( 2.9%) |  |
|  | Claritromicin | 1 ( 1.1%) | 0 ( 0.0%) | 5 ( 3.6%) |  |
|  | Meropenem | 4 ( 4.2%) | 1 ( 3.1%) | 6 ( 4.3%) |  |
|  | Cefotaxime | 3 ( 3.2%) | 1 ( 3.1%) | 12 ( 8.7%) |  |
|  | Linezolid | 0 ( 0.0%) | 0 ( 0.0%) | 6 ( 4.3%) |  |
|  | Teicoplanin | 2 ( 2.1%) | 0 ( 0.0%) | 0 ( 0.0%) |  |
|  | Azitromicin | 1 ( 1.1%) | 0 ( 0.0%) | 0 ( 0.0%) |  |
|  | Ampi/Sulb | 0 ( 0.0%) | 0 ( 0.0%) | 2 ( 1.4%) |  |
|  | Ceftazidime | 3 ( 3.2%) | 2 ( 6.2%) | 0 ( 0.0%) |  |
|  | Ampicillin | 1 ( 1.1%) | 0 ( 0.0%) | 2 ( 1.4%) |  |
|  | Rifampicin | 0 ( 0.0%) | 0 ( 0.0%) | 2 ( 1.4%) |  |
|  | Ceftarolime | 1 ( 1.1%) | 0 ( 0.0%) | 2 ( 1.4%) |  |
|  | Cefepime | 1 ( 1.1%) | 0 ( 0.0%) | 0 ( 0.0%) |  |
|  | Cefazolin | 1 ( 1.1%) | 0 ( 0.0%) | 0 ( 0.0%) |  |
|  | Missing | 2 ( 2.1%) | 0 ( 0.0%) | 1 ( 0.7%) |  |
|  |  |  |  |  |  |
| Atb duration, days |  | 10.0  (6.0-17.0) | 11.0  (4.5-18.0) | 13.0  (9.0-18.0) | 0.075 |

**
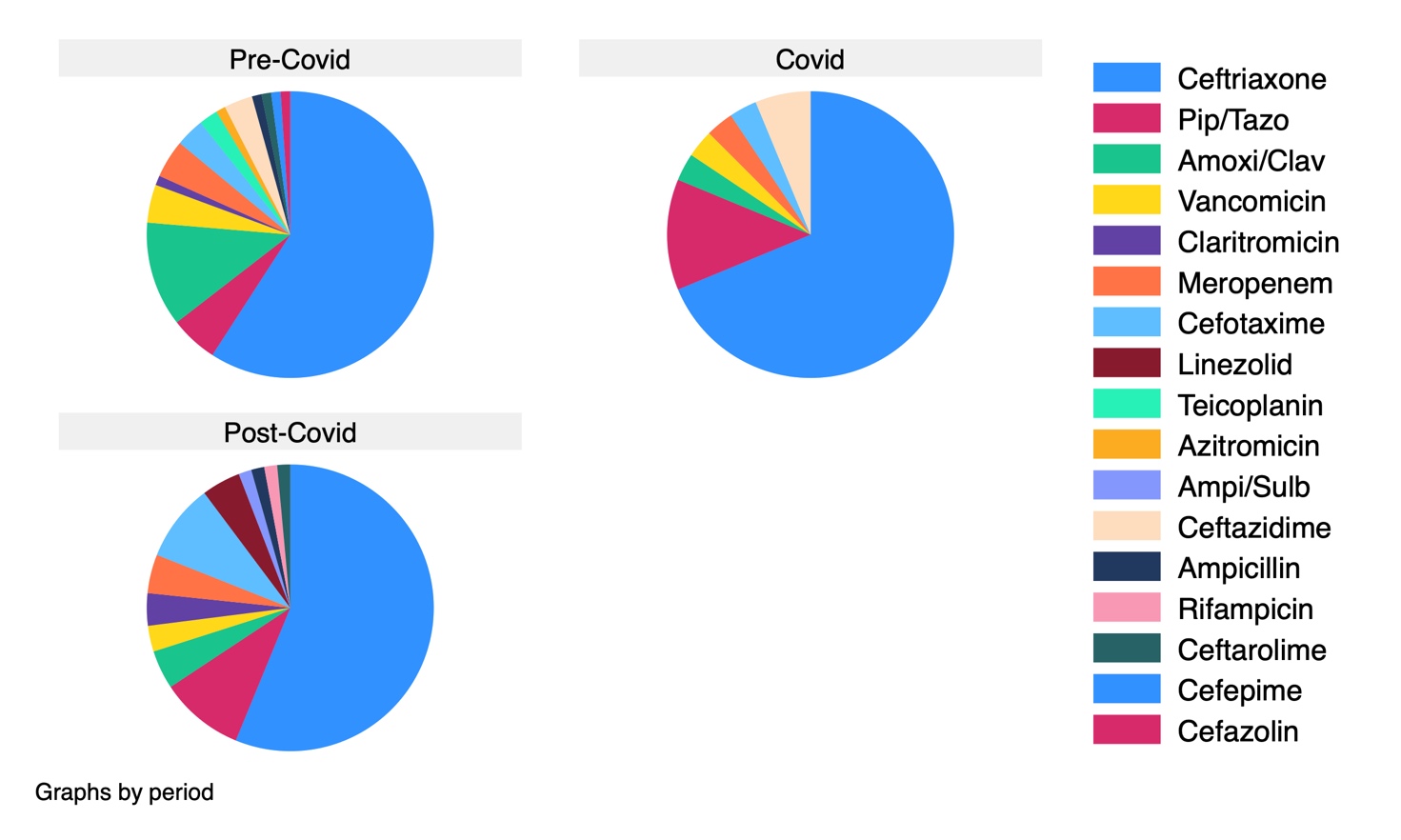
**

Table S2

N of available data per period

|  | Pre (N=95) | Covid (N=32) | Post (N=138) |
| --- | --- | --- | --- |
| Fever days | 87 | 31 | 133 |
| Cough | 94 | 32 | 138 |
| Respiratory distress | 94 | 32 | 138 |
| Chest pain | 94 | 32 | 138 |
| Atb before admission | 94 | 32 | 138 |
| Paracetamol before admission | 64 | 20 | 101 |
| Nsaids before admission | 7 | 2 | 25 |
| Complicated LUS | 77 | 30 | 119 |
| Total antibiotic treatment, median length in days (IQR) | 89 | 31 | 134 |
| Intravenous antibiotic treatment median length in days (IQR) | 87 | 32 | 135 |
| Pleural drainage | 92 | 32 | 137 |
| Fibrinolysis | 89 | 31 | 137 |
| Visual Assisted Thoracic Surgery | 90 | 31 | 137 |
| Surgical resection | 90 | 31 | 137 |
|  |  |  |  |
| Clinical severity | 94 | 32 | 138 |
| Laboratory severity | 94 | 32 | 138 |
| Radiological severity | 94 | 32 | 138 |
| Severe outcome (composite) | 95 | 32 | 138 |
| PICU | 93 | 32 | 137 |
| Oxygen therapy | 93 | 32 | 137 |
| Respiratory support | 69 | 31 | 126 |
| Length of stay | 95 | 32 | 135 |
|  |  |  |  |
|  |  |  |  |
|  |  |  |  |
